# Resisting wh-questions in business coaching

**DOI:** 10.3389/fpsyg.2024.1240842

**Published:** 2024-02-21

**Authors:** Frédérick Dionne, Melanie Fleischhacker, Peter Muntigl, Eva-Maria Graf

**Affiliations:** ^1^Department of English and American Studies, University of Klagenfurt, Klagenfurt, Austria; ^2^Faculty of Education, Simon Fraser University, Burnaby, BC, Canada; ^3^Department of Translation, Interpreting and Communication, Ghent University, Ghent, Belgium

**Keywords:** business coaching, wh-questioning sequences, resistive actions, clients’ resisting, conversation analysis

## Abstract

**Introduction:**

This study investigates clients’ resisting practices when reacting to business coaches’ wh-questions. Neither the sequential organization of questions nor client resistance to questions have yet been (thoroughly) investigated for this helping professional format. Client resistance is understood as a sequentially structured, locally emerging practice that may be accomplished in more passive or active forms, that in some way withdraw from, oppose, withstand or circumvent various interactional constraints (e.g., topical, epistemic, deontic, affective) set up by the coach’s question.

**Procedure and methods:**

Drawing on a corpus of systemic, solution-oriented business coaching processes and applying Conversation Analysis (CA), the following research questions are addressed: How do clients display resistance to answering coaches’ wh-questions? How might these resistive actions be positioned along a passive/active, implicit/explicit or withdrawing/opposing continuum? Are certain linguistic/interactional features commonly used to accomplish resistance?.

**Results and discussion:**

The analysis of four dyadic coaching processes with a total of eleven sessions found various forms of client resistance on the active-passive continuum, though the more explicit, active, and agentive forms are at the center of our analysis. According to the existing resistance ‘action terminology’ (*moving away* vs. *moving against*), *moving against* or ‘opposing’ included ‘refusing to answer’, ‘complaining’ and ‘disagreeing with the question’s agenda and presuppositions’. However, alongside this, the analysis evinced clients’ refocusing practices to actively (and sometimes productively) transform or deviate the course of action; a category which we have termed moving around.

## 1 Introduction

Resisting behavior by clients has received considerable attention in research on psychoanalysis, psychotherapy and beyond (see Fenner et al., 2022 for a recent overview). In psychological discussions, client resistance is framed as an inner or mental phenomenon. It functions as a pertinent feature of the therapeutic process which, while indicating non-complying, opposing or avoiding behavior on the clients’ side, represents an important window to clients’ therapy-relevant thinking and feeling. As such, it should be treated productively as an instrument to work with clients, rather than against them (Safran and Muran, 1996). A growing body of conversation analytic/CA-based research on helping professions (e.g., psychotherapy, counseling) conceptualizes resisting as an interactional phenomenon. Thus, resistance is not an inherent feature of clients, but rather a joint construction between helping professionals and help seekers as they orient to interactional norms and constraints (Muntigl, 2013, 2023, p. 254; see, e.g., Keselman et al., 2018 for therapy; e.g. Peräkylä, 1995; Silverman, 1997 for counseling; or West, 2021 for supervision). The identification and management of resistance as a mental process is thereby considered as embedded in the practices of managing interactional resistance in the process of psychotherapy or other formats (Yao and Ma, 2017, p. 217).

Within CA (and ethnomethodology), resistance is given different conceptualizations that range from ‘narrow’ to ‘broad’ (see Humă, 2023). For example, whereas more narrow descriptions equate resistance with dispreferred actions, such as disagreeing with assessments or refusing requests, that essentially inhibit the progressivity of the sequence (Craven and Potter, 2010), broader conceptualizations see resistance as going ‘beyond the sequence’ to include social and moral aspects (Joyce, 2022). For our study, we adopt a middle ground by viewing resistance as actions that in some way withdraw from, circumvent, or oppose various interactional constraints set up by a prior action (Muntigl, 2013, 2023). These constraints not only involve some requirement to match the design preference indexed in the prior action (e.g., a polar interrogative inviting a *yes* response), but also to the prior topical agenda and different stances (i.e., epistemic, deontic, affective). Thus, a resistive action may orient to one or many of these features / constraints. The current study builds on a body of CA-based research in questioning sequences (Hutchby, 2002; MacMartin, 2008; Muntigl and Choi, 2010; Yao and Ma, 2017), by examining resisting actions in a previously unexplored setting: business coaching. It aims to shed light on clients’ resistive responses to professionals’ wh-questions in systemic-solution oriented business coaching interactions.

From a CA perspective, both systemic solution-oriented business coaching as well as resisting actions (in wh-questioning sequences) in coaching represent novel research foci. Coaching is a helping intervention of intermediate length that transpires, face-to-face or online, in dyadic sessions of one or two hours between a professionally trained coach and a mentally healthy client. Business coaching is a learning and development format that addresses clients’ work-related concerns from a holistic perspective (Greif, 2008; Graf, 2019; Schermuly, 2019). While many different coaching approaches exist, systemic solution-oriented coaching is most widely practiced across the German-speaking coaching market (Middendorf and Salomon, 2017). It is conceptualized as “a co-active, person-centered, process-oriented and solution-focused form of organizational intervention that aims to support clients’ striving toward self-awareness, self-reflexivity and self-regulation (in an organizational context)” (Graf, 2019, p. 25). There is a relatively recent shift in coaching outcome research from proving its overall effectiveness in the context of common success factors, in particular the working alliance (Schermuly, 2019; Molyn et al., 2022), to critically reflecting its negative side effects (see Graf and Dionne, 2021). The quality of the coach-client bond and a possible resistance in or rupture of this working alliance seems to notably influence the emergence and degree of negative side effects in coaching (Ehrenthal et al., 2020, p. 492; see also Schermuly, 2018; Schermuly and Graßmann, 2019; Graßmann et al., 2020). Although Schermuly (2019), among others, discussed them as naturally occurring phenomena in interaction, resistance and ruptures have so far only been investigated via interview data or questionnaires. In contrast, resisting in coaching as locally emerging, sequentially organized phenomena has so far received little empirical attention. To the best of our knowledge, only two CA-based research papers exist, Sator and Graf (2014) and Winkler (2022).

This study addresses this research gap by further investigating clients’ resisting in coaching conversations. More specifically, we focus on how clients display resistance when responding to coaches’ wh-questions as a locally emerging sequentially structured phenomenon. The motivation underlying this focus is twofold. First, based on insights from a current research project on questioning sequences in coaching (Graf et al., 2023), questions are a prolific intervention in coaching.^1^ What is more, wh-questions are frequent in business coaching interactions.^2^ By virtue of their less constricting character, wh-questions allow for a variety of responses to emerge in second position. The following research questions guide our analysis: How do clients in coaching display resistance to answering coaches’ wh-questions? How might these resistive actions be positioned along a passive/active or withdrawing/opposing continuum? Are certain linguistic/interactional features commonly used to accomplish resistance? While we focus more on second positions, i.e., clients’ reactions to coaches’ wh-questions, we also look at third turns and beyond to show how coaches orient to clients’ responses as *resisting*.

## 2 Resisting in interaction

Our approach to resistance is in concert with Humă et al. (2023), who view this phenomenon as an interactional accomplishment. Humă (2023) has identified varying, yet related conceptualizations of resistance with respect to a *narrow* vs. *broad* focus. For our paper, we adopt a view of resistance that lies within this narrow-broad continuum (Glenn, 2003; Muntigl, 2013, 2023; Berger et al., 2016). In our view, resistive responses are taken as actions that contest or avoid the production of an affiliative or aligning response in various ways. Thus, it is not only disagreement, refusal, ‘not answering’ that would count as resistance, but also actions that misalign with a prior speaker’s stance (affective, deontic, epistemic) and delay, defer, or block the trajectory of a certain course of action or interactional project.

One of the central concepts in CA that has gained a lot of currency in explicating resistance is termed *preference organization* (Schegloff, 2007). In responding to a prior action, for example, preference may be characterized as non-equivalent options within a sequence (*preferred* or *dispreferred*) (Schegloff, 2007, p. 58). Preferred responses are generally produced without delay and are ‘pro-social’ in function, often indexing some form of ‘agreement’ or ‘compliance’ with the prior, initiating action (Schegloff, 2007; Pomerantz and Heritage, 2013). Dispreferred responses, on the other hand, are generally *delayed* in their production, signaling a form of disagreement or non-compliance. Dispreferred actions are also *disaffiliative*, which means that they do not work pro-socially and, thus, do not match the projected preference or the affective stance of the prior action (Stivers et al., 2011). Disaffiliation may broadly be seen as a form of non-cooperation with what a prior action is seeking to accomplish, such as disagreeing with a prior assessment, declining a request or not answering a question (Levinson, 1983; Heritage, 1984). Research has shown that dispreferred responses tend to come with certain interaction features, e.g., delaying the production of the response, using mitigating terms, elaborating through accounts, other-initiating repair or using ‘contrastive’ terms (Schegloff, 2007).

Disaffiliation, because it does not offer the ‘preferred’ next action, may be viewed as a form of *resistance*. Here, resistance does not refer to psychotherapy notions involving someone’s conscious or unconscious intentions, but rather to interactional practices that do not support or cooperate with prior action, by not producing an agreement, acceptance, answering the question, and so on. A related term, *disalignment*, also plays an important part in resisting. It refers to actions or conduct that do not move the sequence forward (toward completion) or in some way impede the interactional project underway (Stivers et al., 2011; Steensig, 2020). For example, not taking up a respondent role of ‘empathizer’ to someone telling their trouble would be misaligning because it does not further troubles talk. Not answering a question is also misaligning because the project embodied in the question is momentarily placed on hold. In general, resistance has been viewed as actions or responses that are non-conforming (Stivers and Hayashi, 2010), by not aligning with preferences, topical agendas or stances (epistemic, deontic and affective) and disconfirming presuppositions (see Heritage, 2010).

Resistance has, in the literature, also been viewed in terms of interactional tendencies such as *passive* vs. *active*, which relates specifically to either stalling or directly suspending the progressivity of the interaction (see Joyce, 2022). Eubanks et al. (2015), working within the domain of psychotherapy, conceptualize resistance instead as *moving away* vs. *moving against* (see also Muntigl, 2023). We prefer this conceptualization because we feel it better captures the *action*-orientaton of resisting. Whereas moving away may be more or less equated with *withdrawing*, moving against can be seen as a form of *building opposition* (Goodwin, 1990). A range of withdrawing practices have been identified in psychotherapy interaction: Withholding from responding, acknowledging/weakly conceding, displaying reluctance, denying relevance or validity of someone’s claim. Moving against, on the other hand, is associated with explicit oppositional actions that work to forcefully challenge the constraints of the prior action. Some examples include rejection / disagreement, blame and criticism (see Muntigl, 2023 for a discussion of these forms of resistance in psychotherapy).

## 3 (Resisting in) Questioning sequences

In view of the considerable amount of conversation analytic (or CA-inspired) research on question-answer sequences (e.g., Raymond, 2003; Steensig and Drew, 2008; Tracy and Robles, 2009; and, more recently, Stivers, 2022), little is to be found with a main focus on describing sequences initiated by wh-questions and the types of responses which accompany them, both in mundane or institutional settings such as helping professions. Considering that wh-questions can be implemented in a manner that is less constraining as well as inviting of longer responses (see below), this type of question seems particularly fruitful for (self-)reflection and the co-construction of transformation and change (see Köller, 2004, p. 662), endemic goals across helping professions. Accordingly, the present work contributes to filling this research gap by looking at wh-sequences and systematically describing practices of resistance to answering in the institutional context of business coaching as a helping profession (Graf and Spranz-Fogasy, 2018). In this section, we first review general characteristics of questions, then zoom in on the form of questions under study here, namely wh-questions, before describing established resisting practices associated with this type of question.

### 3.1 Questions

As Hayano (2013, pp. 395–396) states, “questions are a powerful tool to control interaction: they pressure recipients for response, impose presuppositions, agendas and preferences, and implement various initiating actions.” Indeed, as initiating actions questions make answers (or, at the very least, some type of response) conditionally relevant (Schegloff, 2007). In asking them, speakers communicate their assumptions or presuppositions and these, in turn, may be corrected by the recipient with varying consequences for the progressivity of the sequence (Stivers and Robinson, 2006). Clayman and Heritage speak of presuppositions’ “depth of embeddedness” (Clayman and Heritage, 2002, p. 204): if it is impossible for recipients to refute the assumptions contained in the question while still answering, one might speak of deeply embedded presuppositions. Here, recipients must decide whether to ‘simply answer’ and thus accept the presuppositional content of the question, or to modify or reject them, but in doing so avoid answering the question as it was stated (Hayano, 2013, p. 402; see also ‘transformative answers’ by Stivers and Hayashi, 2010). Beyond conveying presuppositions, questions also set both a topical and an action agenda, which convey certain preferences as to what the response should do and contain, as well as how broad or precise the response to the question might be (*cf.* Clayman and Heritage, 2002; Hayano, 2013, p. 403). Specific question forms also contain preferences regarding how they should be formulated: among others, there is a preference for answers (vs. non-answers such as no-access claims, or a lack of reaction altogether; *cf.* Stivers and Robinson, 2006; Hayano, 2013, p. 404) and one for type-conformity (vs. non-conformity; *cf.* Raymond, 2003; Hayano, 2013, p. 407).

### 3.2 Wh-questions

In light of the breadth of the phenomenon “question-answer sequence,” we focus on one specific form, namely wh-questions. Following Couper-Kuhlen and Selting, we define wh-questions as interrogatively marked utterances which make use of ‘question words’ to request specific kinds of information: the who, what, when, where, how and why of a given situation or state of affairs” (Couper-Kuhlen and Selting, 2018, p. 20). Wh-questions are accordingly most frequently heard as requesting information from a lower epistemic stance (K-) perspective (Yoon, 2010; Heritage, 2012; see also Couper-Kuhlen and Selting, 2018, p. 221) and as such make the delivering of the sought-after information in an answer relevant. Within this relevance constraint, wh-questions are characterized by a general open-endedness as regards answer possibilities, which may be modified by using prefaces (Clayman and Heritage, 2002, p. 201). A major feature of wh-questions is their propitiousness for deeply embedded presuppositions (*ibid.*, p. 206). According to MacMartin (2008), this last feature makes them a particularly thought-provoking intervention in helping interactions. However, little research has been done so far focusing on wh-questions and questioning sequences in these (institutional) contexts.

In psychotherapy, MacMartin (2008) investigated optimistic questions, defined as wh-interrogatives that “prefer answers from clients that affirmed their agency, competence, resilience, abilities, achievements, or some combination thereof” (MacMartin, 2008, p. 82). Though designed to secure client cooperation, MacMartin found client disaffiliation with the optimistic agenda remained a possibility (see below). Mack and colleagues, in 2016, published an investigation of verb-first and wh-questions occurring in four German-speaking first psychotherapeutic interactions. Exploring whether questions may fulfill the same four functions that formulations do (see Weiste and Peräkylä, 2013), they found wh-question forms to do mostly highlighting and rephrasing actions. Beyond this, Mack and colleagues’ study also found two other functions of questions: collaborative explanation-finding questions (Mack et al., 2016, p. 86) and solution-oriented questions (*ibid.*, p. 81). While the former made use of both types of interrogative syntax, the latter was mostly designed using wh-questions. Kabatnik et al. (2019) also focused on solution-oriented questions and found that clients’ responses were mostly dispreferred or insufficient.

As already indicated above, (wh-)questions in coaching have remained largely unexplored from a CA perspective, with only a few studies reporting on comparative findings between coaching and psychotherapy (e.g., Spranz-Fogasy et al., 2019 with example requests; Kabatnik and Graf, 2021 with solution-oriented questions). This existing research has not yet focused on the format of the question, but rather investigates particular (functional) question types and their interaction-specific sequential development following Peräkylä’s (2019) model of transformative sequences.

### 3.3 Resisting in the context of wh-questioning sequences

Thompson et al. (2015) provide a systematic examination of the breadth of possible linguistic forms which occur in recipient turns to wh-questions from a discourse-functional/interactional linguistic perspective. Basing their findings on mundane interactions occurring in English, they distinguish two types of wh-questions that set different kind of relevancies: “Specifying Questions seek single, specific pieces of information. Telling Questions, on the other hand, seek extended responses – reports, stories, accounts, explanations, and so on” (Thompson et al., 2015, p. 20). On this basis, they identify three response types for wh-questions: phrasal responses, expanded clausal responses, and unrelated clausal responses, which in their mopho-syntactic form index problems with the initial question, e.g., expanded clausal responses to Specifying questions.

While Thompson, Fox and Couper-Kuhlen provide an overview of the grammatical forms that responses to wh-questions may take, MacMartin (2008) offers further insights into responses to optimistic (wh-)questions indicating trouble in psychotherapy sessions. She investigates the strategies used to resist and thus disalign and disaffiliate with the optimistic agendas contained in wh-questions (made difficult by the pesuppositions’s depth of embeddedness) and distinguishes two main types of resisting responses: answer-like and non-answers. Answer-like responses include optimism downgraders, joking or sarcasting responses, and refocusing responses, which move the focus away either from the optimistic dimension or attribute it to external factors. Non-answers represent more explicit forms of resisting and disaffiliating in that clients openly position themselves as unable or unwilling to engage with the optimistic agenda of the questions (MacMartin, 2008, p. 89) via complaining, or refusing to cooperate with elements of the question, e.g., some presuppositions.

In the context of coaching, Sator and Graf (2014) tackle resistance in connection with knowledge management and more specifically, with (dis-)aligining forms of client participation in (re-)structuring knowledge within question-answer sequences. Their analysis focusses on one coaching session and investigates both the thematic contexts of the client’s resistance as well as the sequential organization of interactional trouble. Winkler (2022) explores ‘semi-responsive answers’ to all types of questions. The study applies a (CA-based) coding scheme for (semi-)responsive answers following criteria pertaining to the topical dimension (e.g., topical shifts and expansions to additional topics, topical narrowings, refusing to engage with the agenda) and formal dimension (e.g., shifts in perspectivation and verb tense as well as use of mitigating strategies) (Winkler, 2022, pp. 159ff).The focus of the analysis lies on degrees of responsiveness in client answers as well as on categorizing coaches’ reactions to these in third positions.

Previous research has centered on resistance in the context of a particular (thematic-functional) question type and within question-answer sequences in general. Though categories for semi-responsiveness have been introduced by Winkler (2022) and MacMartin has distinguished dis-aligning / dis-affiliative responses to wh-questions, no systematic conversation analytic investigation of resistive answers to wh-questions has so far been carried out for business coaching. In our contribution, we build on previous findings but describe the variety and extent of resisting in recipient turns, thereby paying attention to interactional tendencies on the passive vs. active or ‘moving away’ vs. ‘moving against’ spectrum previously identified in other helping formats.

## 4 Data and methods

### 4.1 Data

The data for this study stem from a larger corpus of systemic-solution oriented business coaching interactions that were collected between 2021 and, 2023 for the international and interdisciplinary research project *Questioning Sequences in Coaching* (QueSCo–Questioning Sequences in Coaching, 2023).^3^ The coaching processes were audio- and video-recorded by the coaches and subsequently minimally transcribed following cGAT2 conventions (Schmidt et al., 2016). The extracts included here were then adapted to reflect conversation analytic conventions (e.g., Hepburn and Bolden, 2013).

For the present study, we randomly selected four dyadic coaching processes with two to three sessions each, which amount to approximately 13 h of coaching interaction. The dyads include different coaches and clients; the first process, CO3-KL1, takes place between a female coach and a female client; the second, CO7-KL1, occurs between a male coach and a female client; the third process, CO9-KL1, has a male coach and a male client; finally, the fourth process, CO10-KL1 involves a female coach and a male client. Whereas CO3-KL1 and CO10-KL1 occurred in face-to-face setting, both CO7-KL1 and CO9-KL1 took place online. Though the coaches all work within the systemic solution-oriented approach, their procedure displays idiosyncratic features. The variation of the data aims to demonstrate that clients’ resisting practices are not specific to particular coaching approaches and relationships, but can be identified across different processes.

### 4.2 Method

For the purpose of this study, we drew on the methods of Conversation Analysis (CA). CA aims “to identify structures that underlie social interaction,” and thus to detail “the intertwined construction of practices, actions, activities, and the overall structure of interactions” (Stivers and Sidnell, 2013, p. 2). This is based on the ethnomethodological premise that participants share practices of reasoning that they use to make sense of each other’s actions, and because these practices are enacted in conversation, they can thus be systematically described (Heritage, 2001). To do so, conversation analysts look at sequences of talk to determine how participants accomplish actions, convey meaning, and display understanding both from an initiating and recipient perspective. Accordingly, a speaker who initiates an action such as a request for information can be understood as doing so on the basis of a shared common-sense knowledge of what a request for information ‘is’ and ‘does’; the recipient, in turn, will show their understanding of the speaker performing this action by, for example, providing the sought-after information made relevant by the initial request. In cases in which recipients do not orient to the initial speaker’s talk as requesting information, repair might be initiated by the latter to re-establish a mutual understanding–i.e., intersubjectivity–of what is currently being pursued in the conversation (see, e.g., Kitzinger, 2013). All in all, this means that knowledge and understanding but also social relations are co-constructed and indeed updated on a turn-by-turn basis in conversations through the participants’ mutual orientation.

On this basis, CA has gained particular ground in the field of helping interactions as it enables the tracking of change as it develops through the means of sequential analysis. Indeed, as Peräkylä (2019, p. 267) convincingly argues, transformation can be documented within sequences, as referents, emotions, and relationships are updated turn-by-turn and by the same move modified to some extent by the speakers. Close sequential analysis, then, can illuminate the process through which ways of thinking and feeling about actions, events etc. are changed, new knowledge is shared and acquired, and relationships are negotiated and nurtured (*ibid.*). In the same way, ambivalence and difficulty in these tasks can be observed by looking at sequences of talk in which the recipient resists some or all aspects made relevant by the initating action (see, e.g., Voutilainen et al., 2011 and various works by Muntigl et al. on psychotherapeutic interactions). Uncovering the practices through which such resistance is manifested is an endeavor which we undertake here in the context of coaching interactions.

### 4.3 Procedure

The first step consisted of gathering all questioning sequences with interactional trouble (in the sense of sequences with dis−/misaligning and/or disaffiliating reactions) in the clients’ responding turns from the transcripts and the recordings of all selected sessions. As the data used for this study was collected for the project *Questioning Sequences in Coaching*, questioning sequences had already been determined. The first round of analysis led to a discussion as to what may be considered ‘resisting’ in coaching, taking prior work on resistance (in questioning sequences and in other professional formats) but also the specificity of the interaction into account. Considering the wide array of possibilities these questions offer to clients for responding, the focus on wh-questions was established.

In a next step, wh-questioning sequences which displayed similar resistive actions in the second pair part (e.g., remaining silent, modifying question’s terms or invalidating the coach’s course of action through a limitation of agreement) were grouped into preliminary categories thereby inductively carving out relevant (categorization) criteria and features for resistive responses in coaching. These criteria were then used to re-analyze the entire data in a second round of identification: all sequences initiated with wh-questions in the four selected processes were again systematically verified for these markers of resistance. This yielded a collection of 82 wh-questioning sequences containing all practices of resistance on the active/passive or explicit/implicit continuum; this also included ‘no response’, ‘minimal acknowledgement’, ‘initiating (other-)repair’ and ‘accounting (for not answering)’, which function as ‘moving away’ or ‘withdrawing’ practices. However, since these phenomena have already been dealt with extensively in existing conversation analytic literature (see Muntigl, 2023 or Humă et al., 2023 for a recent overview), they will not be further discussed in the present work. Table 1 presents the distribution of the all resistive sequences according to the coaching process and session.

**Table 1:** Distribution of sequences displaying client resisting actions across processes and sessions.

Process	Session 1	Session 2	Session 3	Total
CO3-CL1	12	10	7	29
CO7-CL1	5	5	9	19
CO9-CL1	6	2	3	11
CO10-CL1	14	9	*n.a.*	23
All processes				82

The following section presents the results of our analysis of the remaining wh-sequences, detailing their distinct features and illustrating these with examples.

## 5 Findings

Overall, we found that a large majority of sequences initiated by a wh-question in our data (indeed 219 out of a total of 303 wh-questioning sequences; i.e., in average approximately 73% per process) develop without clients resisting in their recipient turn; resisting occurs in about 1/5 to 1/4 of the wh-questioning sequences within an entire coaching process. Accordingly, this might showcase a tendency for affiliation by clients with their coaches, and, by the same move, strong personal engagement in their coaching project, i.e., change and development–at least in respect to this particular questioning sequence type.^4^

In the analysis of wh-sequences displaying resistance, consideration was given to the relative strength of the resistive responses in terms of whether the progressivity of the ongoing course of action was suspended or not and whether clients performed resisting while or without responding (Humă et al., 2023). We found practices that can be attributed to the previously established category of ‘moving against’ or ‘opposing’, in which clients resist or “push back against” (Humă et al., 2023) the question constraints by overtly disagreeing with presuppositions, or the plain asking of a (wh-)question thereby (actively) opposing or blocking the smooth progression of the wh-questioning sequence. Subtypes include ‘refusing to answer’, ‘complaining’ and ‘disagreeing with the question’s agendas or presuppositions’.

However, we have also identified client practices that work to change, transform or deviate the question’s course of action in more cooperative ways, thereby establishing a middle ground between ‘moving away’ and ‘moving against’. Clients sidestep the question’s constraints, i.e., the suggested trajectory of the coach, but do not (entirely) block the progressivity of the sequence. This means that the overall coaching project may move forward regardless of the non-compliance with the suggested action. We have assigned them to a third category, i.e., ‘moving around’ or ‘refocusing’. Clients’ refocusing thereby includes circling or ‘looping’ back to the underlying problem or from inner states to external contextual factors, but also the introduction of alternative solutions or topics than those introduced by the coach. We have found instances of refocusing with or without a preceding (pro-forma / partial) answer (see Table 2 for an overview of the distribution of the number of instances for these (sub-)categories).

**Table 2:** Distribution of instances for each resistive reaction (sub-)category.

Type	CO3	CO7	CO9	CO10	Total
**Moving against/Opposing**					
Refusing to answer	0	0	0	1	1*
Complaining	0	2	0	0	2*
Disagreeing with question’s agendas and presuppositions	3	2	3	1	9
**Moving around / Refocusing**					
Not answering and refocusing	1	1	0	4	6
(Partial) answering but refocusing	2	3	2	1	8
Total	6	8	5	7	26

*Even though the number of instances for these categories is low, they (must) constitute possible forms of resistance.

In our findings below, we first present examples for each of the subtypes of ‘opposing’ (organized according to decreasing displays of client resistance), and then turn our attention to the ‘refocusing’ subtypes, which constitute the categorical novelty introduced in this paper.

### 5.1 ‘Moving against’: opposing

‘Moving against’ in the sense of opposing (part of) the constraints contained in the wh-question is realized through three subtypes, namely ‘refusing to answer’, ‘complaining’ and ‘disagreeing with the question’s agendas and presuppositions’.

#### 5.1.1 Refusing to answer

Unlike its non-verbal counterpart, remaining silent, which may index a disengaging (i.e., a *withdrawing*) form of resistance, a verbalized refusal to answer constitutes a strong form of explicit opposition by the client to the coach’s question and the suggested course of action embedded in the wh-question. It blocks the progressivity of the sequence and marks a possible rupture in the working alliance between coach and client (Muntigl, 2013). Extract 1 displays this form of ‘opposing’. The sequence under study follows a questioning sequence that topicalized an ideal coaching outcome to the client’s problem of being overworked. This was first met by silence and – after the coach produced various (explanatory) increments–a counter-question from the client inquiring about the coach’s knowledge of the “Serenity Prayer.” Using said prayer to structure his response, the client alludes to a wish of being able to differentiate between things that he can and cannot change (data not shown).

**EXTRACT 1:** Refusing to answer.

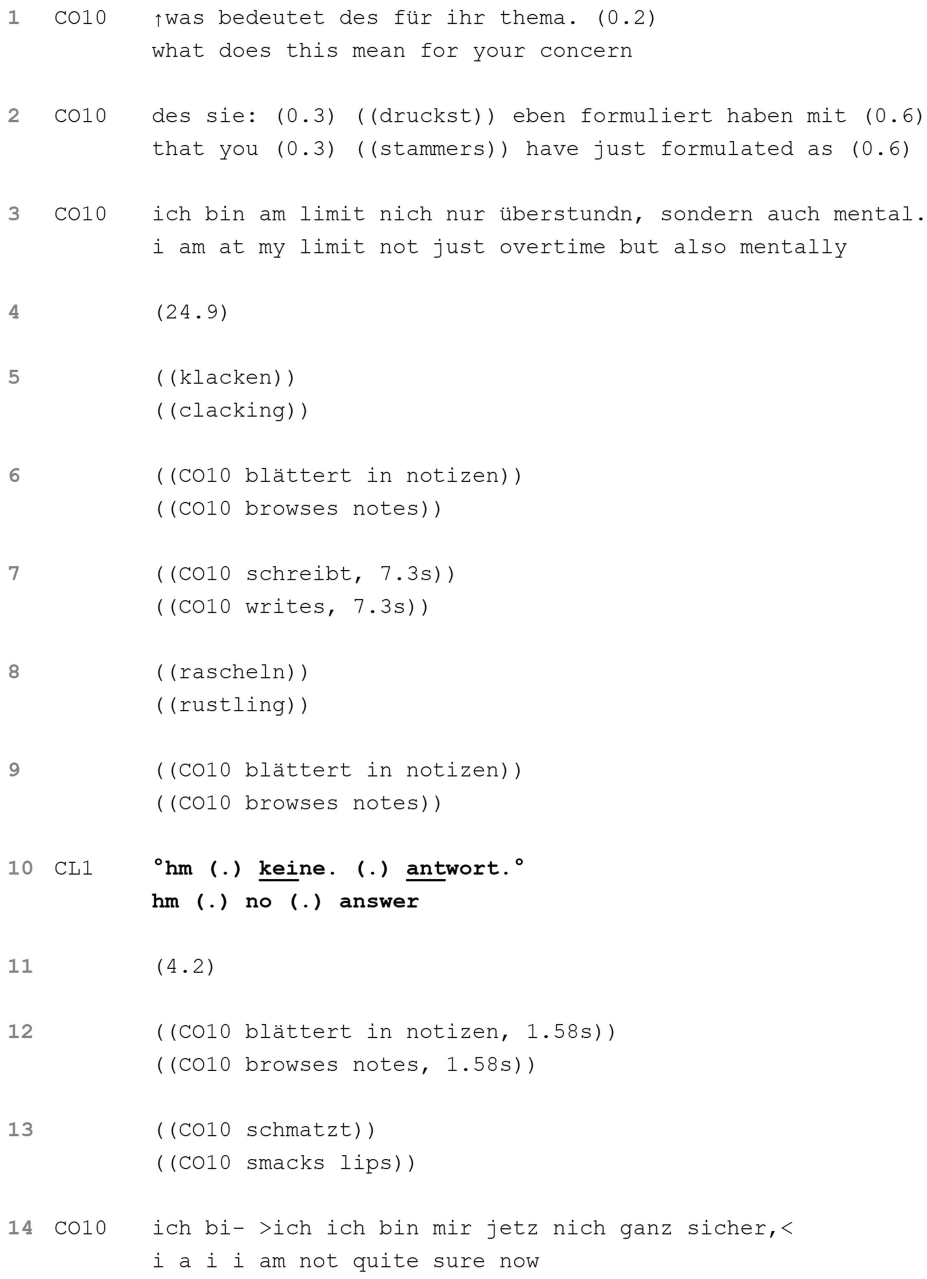

Since the ideal coaching outcome made relevant by the coach’s former question remains unclear, the coach follows up with the question “what does this mean for your concern” (line 1), making a connection to the client’s initial concern conditionally relevant. The asking is in itself mildly disaffiliative, perhaps implying that the client has been talking off topic. Since the client does not take up speaking rights at the next transition-relevant place, the coach further explains her meaning in increments (Schegloff, 2016; lines 2–3), thus insisting on the relevance of a response by the client in relation to his previously formulated concern. Following this, an extremely long gap (24.9 s) ensues in line 4, only intermittently interrupted by the coach’s reviewing and completing her notes. By withholding from taking back speaking rights, she signals that she expects at least some form of engagement from the client.

The client finally produces a verbal response in the form of a short acknowledgment token, a micro-pause and an explicit refusal to engage with the question (“no answer”) (line 10). In doing so, the client fully and explicity stops the progressivity of the course of action (Joyce, 2022), both disaligning by producing a non-answer and disaffiliating by opposing the coach’s project and disregarding her insistence for a response. Beyond this, the act of refusing to answer a question and baldly saying so is threatening to social cooperation and therefore the coach-client relationship. Another silence emerges (4.2 s), with the coach consulting her notes and in which the client does not provide an account for his refusal to answer. As the coach reclaims speaking rights, her turn begins with cutoff speech and an admission of uncertainty or insecurity (line 14).^5^

#### 5.1.2 Complaining

In this subtype, clients express trouble with the wh-question by complaining. They voice some (moral) indignation or dissatisfaction *about* or *to* the coach, e.g., for asking the question in the first place or about the difficulty of the question (MacMartin, 2008) and, thus, they direct criticism toward the coach and/or coaching process. In this way, a complaint sequence gets initiated instead of answering the question. Complaints as first pair parts do not have typed second pair parts, but may be followed by, for instance, offering a remedy, denial, justification, rejection, excuses, or acceptance (Laforest, 2002; Schegloff, 2007; Couper-Kuhlen and Selting, 2018). Since complaints are potentially face-threatening and as such usually formulated indirectly, it is up to the recipient to decide whether their behavior is being reprimanded (Laforest, 2002; Pomerantz, 2021). Because clients do not orient to the question in a productive way, thereby suspending the conditional relevance of the question and blocking the progressivity of the sequence, complaining constitutes non-answering and thus a more direct form of resisting the asking of the question. In Extract 2, after having spent the first 15 min of the session on extensive problem exploration, the coach first summarizes his client’s concerns and then invites her to select one of these issues as a focus for the session. This is the coach’s second attempt at inviting the client to set a goal; however, at the beginning of the session the client was unable to do so. Yet again, the client expresses trouble or reluctance to select a focus by complaining.

**EXTRACT 2:** Complaining.

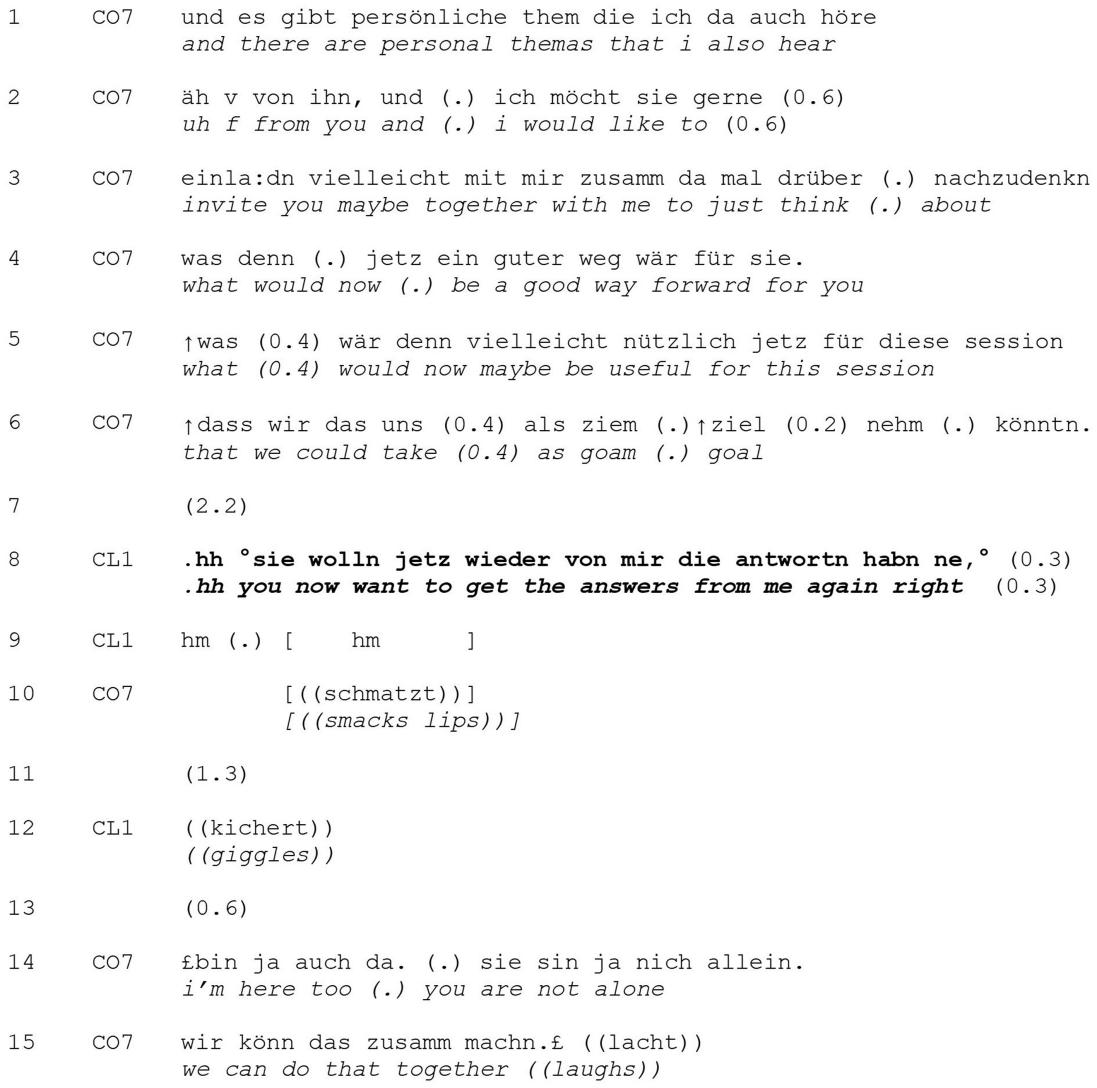

In lines 1–2, the coach finishes a multi-turn formulation of the client’s concerns supporting it via evidential markers (“that I also hear from you”). This is followed by a proposal from the coach to start thinking about a potentially helpful way forward (lines 2–6). While the coach makes use of his deontic right to suggest a subsequent action (see Jautz et al., 2023.), this is mitigated via his offer of support (“maybe together with me”). He immediately follows up on his request with a wh-question asking the client to select a suitable goal for their session (“what would maybe now be useful for this session”).

After a 2.2 s turn-initial delay, the client breathes in audibly before uttering her complaint (in line 8), which teasingly expresses her dissatisfaction with her role as questionee, i.e., about being “forced into a discursive role” (Muntigl, 2023, p. 293) and the “requirements or constraints placed upon [her] mode of conduct” (*ibid.*, p. 292). The use of the adverb “again” constructs this as a repeated activity. Her assertive utterance, which functions as a non-answer, is followed by a question tag “right” inviting agreement. Since complaints threaten social cooperation (Laforest, 2002), the client then produces acknowledgement tokens (line 9) suggesting reflection and, after a 1.3 s gap in line 11, starts giggling. In doing so, she signals that her complaint should be understood as a joke, thereby constructing a ‘non-serious’ frame and minimizing the threat to the coach’s face. In his response, the coach makes himself available in his supportive role, reducing the pressure on the client as the sole person responsible for finding answers. He then repeats his invitation to work collaboratively, constructing coaching as a conversation at eye level (Jautz et al., 2023) and echoes the client’s affiliative laughter first in responding in a ‘smiley voice’ (lines 14–15) and then in joining in (line 15).

The client’s complaining response in Extract 3 is designed similarly. Earlier in the session, the client had explained that she generally has difficulties staying “in the moment” and tends to think of the future instead (data not shown). Just prior to the sequence, the coach and the client have been discussing various motivations and strategies she uses to help her focusing on the present. After the client has already named a few, the coach asks for further strategies (line 1).

**EXTRACT 3:** Complaining.

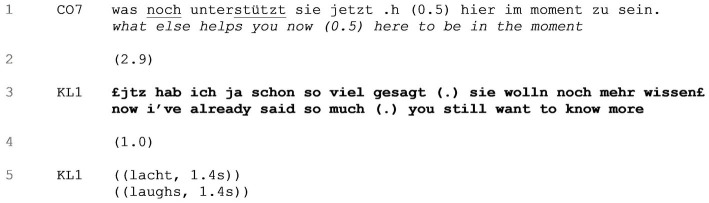

Following an initial silence of 2.9 s in line 2, in a smiling voice, the client criticizes the coach’s request to further elaborate. She claims that she has “already said so much” (line 3) and lightheartedly accusing the coach of being still unsatisfied with her cooperation (“you still want to know more,” line 3). A 1.0 s gap ensues as the coach withholds from taking a turn (line 4)^6^ and then the client finally starts outright laughing outright here again (line 5). As in Extract 2, she pushes back against the constraint of having to answer at all, and by the same move demonstrates (good-humored) opposition to the simple asking of the question.

#### 5.1.3 Disagreeing with the question’s agendas and presuppositions

In the subtype “disagreeing with the question’s agendas and presuppositions,” clients problematize the question’s formulation and/or the presuppositions contained therein, i.e., they problematize a part of the prior action. In line with Clayman and Heritage’s (2002) as well as MacMartin’s (2008) findings on responses to questions with deeply embedded presuppositions (i.e., wh-questions), we generally found an explicit refutation of these. In contrast to the first two ‘opposing’ categories, though clients refrain from answering the initial wh-question, they might be working toward changing the embedded presupposition so as to answer a (slightly) different question or provide material for the coach to adjust their question or initiate another intervention (e.g., a follow-up question as in Extract 1). In other words, clients may respond in a way which may allow the coaching project to progress although retroactively modifying the coach’s initial question (similar to transformative answers to polar interrogatives, see Stivers and Hayashi, 2010). In our first example (Extract 4), coach and client had previously been discussing the client’s reported inability to remain or return to a more serene state in the hectic of her work life. The extract sees the interactants exploring the relationship between the client’s ‘hectic’ and ‘serene’ states.

**EXTRACT 4:** Disagreeing with the question’s agendas and presuppositions.

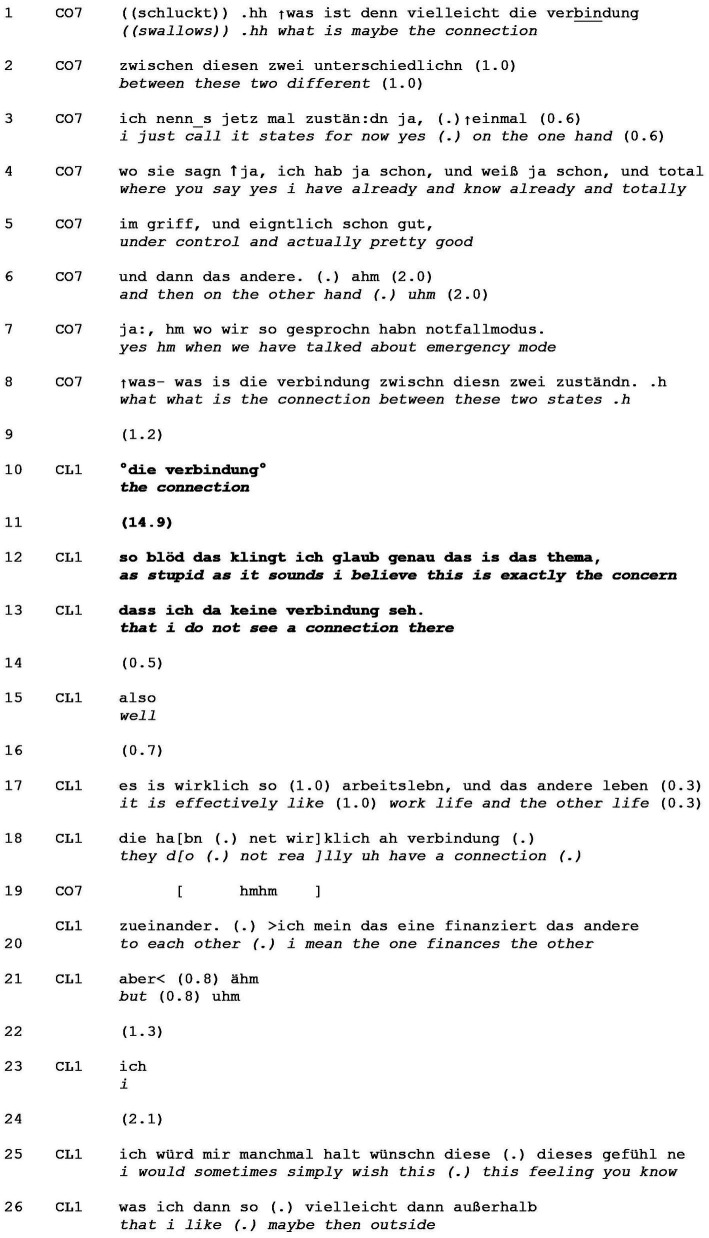

In line 1, the coach starts formulating a question before aborting to search for the right expression, which he metapragmatically comments on “i just call it states for now yes” (line 3). Referring back to what had been discussed so far, the coach elaborates on “these states”. Having now set the context for his question (Clayman and Heritage, 2002), the coach reiterates his initial question. By making a reflection on the nature of the “connection” conditionally relevant, the coach presupposes that there is such a link. It is precisely this presupposition that the client then identifies as problematic.

After a gap that already indicates probable misalignment (line 9), the client repeats the core element of the presupposition (“the connection,” line 10). By means of this partial repeat, the client *mirrors* an aspect of the coach’s prior talk (Ferrara, 1994), which not only functions as a request for elaboration but also possibly locates this element of the question as repairable (Schegloff et al., 1977; Robinson and Kevoe-Feldman, 2010) and suggests a divergence of views and impending disagreement. This is also in line with earlier findings, in which repetition is indicative of resisting (Peräkylä, 1995, p. 279; see also Heritage and Raymond, 2012). A 14.9 s gap (line 11) ensues, in which the coach does not engage in elaboration nor in self-repair, and indeed withholds from responding altogether, thereby implicitly “insisting” on his question, i.e., the presupposed “link” between the client’s states. This puts pressure on the client to reflect and formulate her own thoughts on “the connection” problem, i.e., to solve the issue (see also Muntigl et al., 2020b for psychotherapeutic interactions).

Following the coach’s declining to take a turn, the client disagrees with this deeply embedded presupposition, thereby veering into non-answer territory (MacMartin, 2008). To mitigate, she prefaces this with a deprecating disclaimer (“as stupid as it sounds,” line 12) and frames her explicit refutation of the presupposition in line 13 as the problematic element that she in fact needs to address. She adds precision to this by highlighting her perceived disconnection of work life and private life with the adverbs “effectively so.” The preface “well” also constitutes “an alert to the non-straightforwardness” to follow (Schegloff and Lerner, 2009, p. 102) and suggests a resistance to the question’s project (Muntigl, 2013). The turn-final conjunctional “but” in line 21 serves as a “trailoff” (Schegloff, 1996) allowing speakership transition at a pragmatic but not syntactic turn-completion. The client thereby indicates a “possible action completion for ‘contrasting’ that has been constructed in the current and prior courses of action” (Hata, 2016, p. 139).

Extract 5 is another example of the client retroactively modifying the question’s agendas and presuppositions. The sequence takes place shortly after the client has finished reporting about a recent job interview, which had left her disappointed. The client has wondered whether she should be less “demanding” in terms of criteria for the positions she applies for, which prompts an exploration about how she could have been less demanding, and then, as shown in the extract, why the client feels that way (lines 1–2):

**EXTRACT 5:** Disagreeing with question’s agendas and presuppositions.

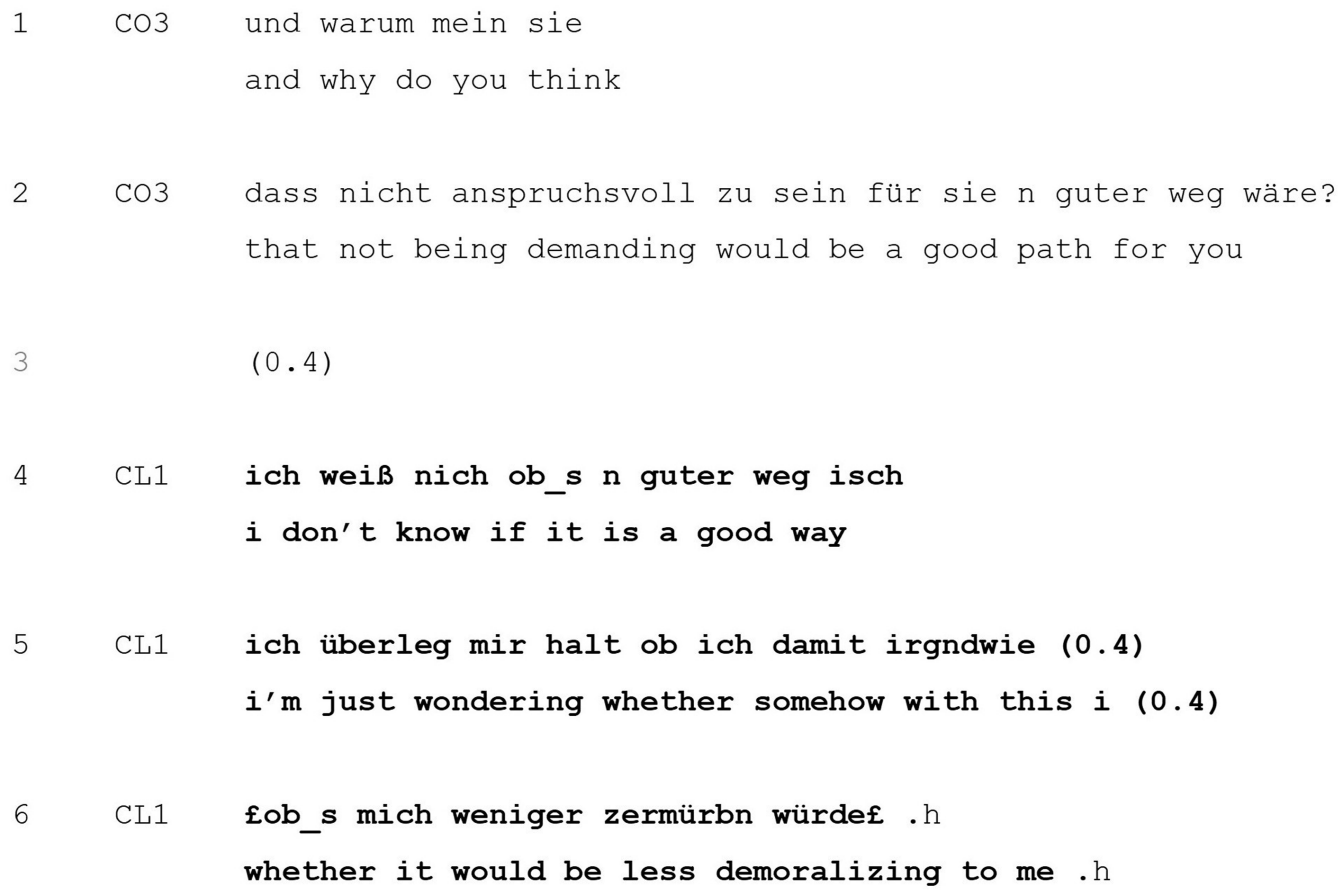

With the wh-question, the coach conveys the presupposition that the client believes that such a change in behavior (i.e., being less demanding) would be “a good path” for her (Extract 5, lines 1–2). By virtue of being a why-question, the interrogative here can double as both a genuine request for an explanation and as a challenge to the client’s possibly problematic belief (Bolden and Robinson, 2011). It is to this assumption that the client first orients to in her response: she first refutes the presupposition in correcting that she is unsure whether it would be “a good path” (line 4). In doing so, the client disagrees with the question’s agendas and presuppositions. Instead, she offers an alternative explanation to the coach’s erroneous assumption, namely that it would possibly be “less demoralizing” to her (line 6). Thus, resisting the coach’s challenge of a positive perception of being less demanding, the client adjusts the question and maintains her framing of it as an alternative solution which may have a more positive outcome.

### 5.2 ‚Moving around’: refocusing

In this category, clients move around the coaches’ initial course of action and refocus on their own. They may do so all the while engaging with the question in some manner, for instance by answering in what then reveals itself to be a pro-forma manner, or they may also pursue their own alternative course of action right away.

#### 5.2.1 Not answering and refocusing

In this subtype, clients do not provide a (partial or pro-forma) answer in their responding turn and solely refocus the course of action. At times, this is due to the deep embeddedness of the presuppositions. The refocusing may take place on various levels, as Extract 6 shows. It follows the description of a problematic situation in the client’s work environment. The client had complained that a colleague refused to follow the standard procedure for looking up information, turning to his team instead. This eventually resulted in the colleague insulting him as a “know-it-all.” In spite of the client’s report of the incident to their supervisors, the colleague faced no consequence.

**EXTRACT 6:** Not answering and refocusing.

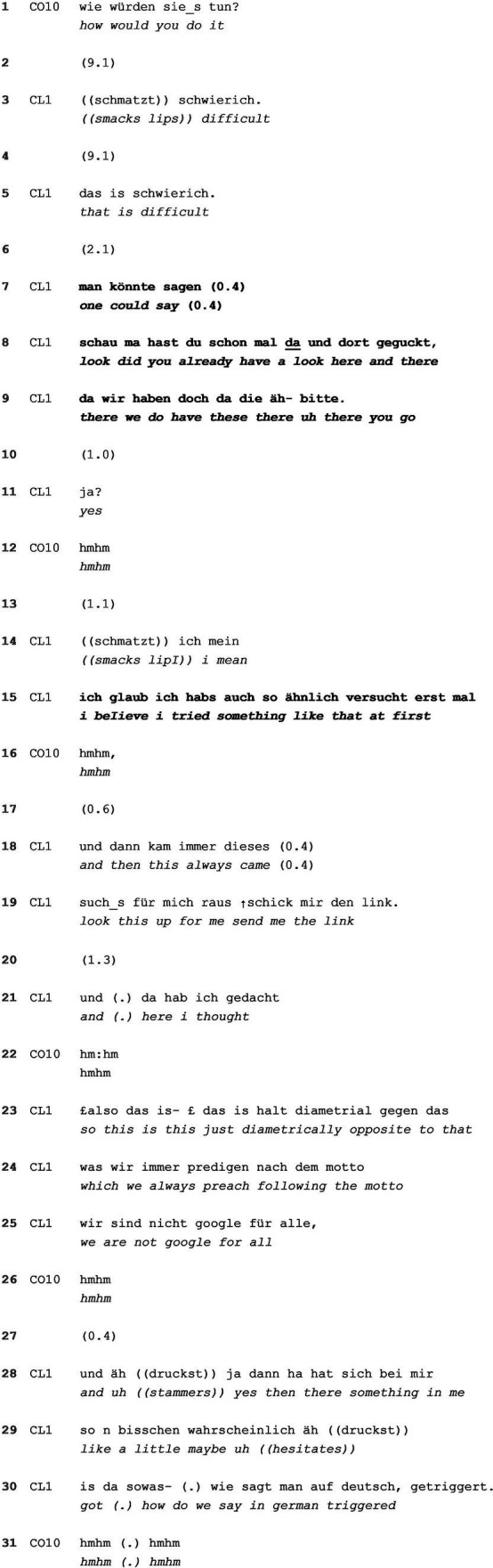

After the client shortly brings up the possibility of remaining silent, which is immediately rejected as an appropriate alternative behavior, the coach asks the hypothetical wh-question under study here: “suppose you would come to be in such a situation again and you would shape it in the best way possible for yourself, how would you do it?” (data partly shown, line 1). By doing so, the coach makes an ideal solution, i.e., a hypothetical, ideal scenario in which the client could adapt his own behavior, thinking or feeling in any imaginable way, conditionally relevant in the responding turn.

Upcoming disalignment from the question is foreshadowed by the 9.1 s silence in line 2, an evaluation of the question as “difficult,” i.e., troublesome to answer (line 3), and the repetition of this in line 5. The rise-fall contour of the first evaluative “difficult” is striking here and might point to the speaker’s contrasting or conflicting attitude regarding the question (Zahner-Ritter et al., 2022). Nonetheless, the coach withholds from taking turns. After another 2.1 s gap in line 6, the client formulates a possibility using the impersonal, no-agent pronoun “man” (translated as “one” here; lines 7–9), thereby distancing himself from the solution as being ideal for him and speaking from a more general position. The imagined alternative remains quite vague and does not index any “best” or more suitable way to deal with such a situation. This is the first element of refocusing, i.e., the client refocuses the solution orientation away from himself as the agent circumventing the question’s constraints. This results in a 1 s silence in line 10, leading the client to explicitly indicate that he has concluded his turn in line 11 with “yes.”

In response to this, the coach produces minimal ratification (line 12), which in combination with the 1.1 s silence in line 13, prompts the client to continue with an elaboration. From there on, the client further refocuses away from the conditionally relevant solution orientation and brings back the problem orientation by accounting for his previous reaction and referring to common practices within his department. The client’s account also displays elements of verbosity (Fenner et al., 2022) as indicators for resistance, such as directly quoted dialog, re-counting the problematic situation in detail, a focus on third parties, and emotional distancing. Again, the client steers away from the coach’s solution-oriented interactional project of “describing the client’s ideal alternative behavior” suggesting a need for further problem-orientation.

In some cases, however, clients do not answer and (partially) refocus on solution-orientation, as the next example shows. Extract 7 begins shortly after coach and client have set the goal that the client wants to feel more self-confident in her abilities and generally more serene. For the moment, she still lacks confidence and tends to reconsider her every action “twenty thousand times” (data not shown). The coach then focuses on the ideal state of the client and requests her to name example situations in which she had already been successful in achieving self-confidence and serenity in the past (Extract 7, lines 1–7) (see Spranz-Fogasy et al., 2019 for working with example situations).

**EXTRACT 7:** Not answering and refocusing.

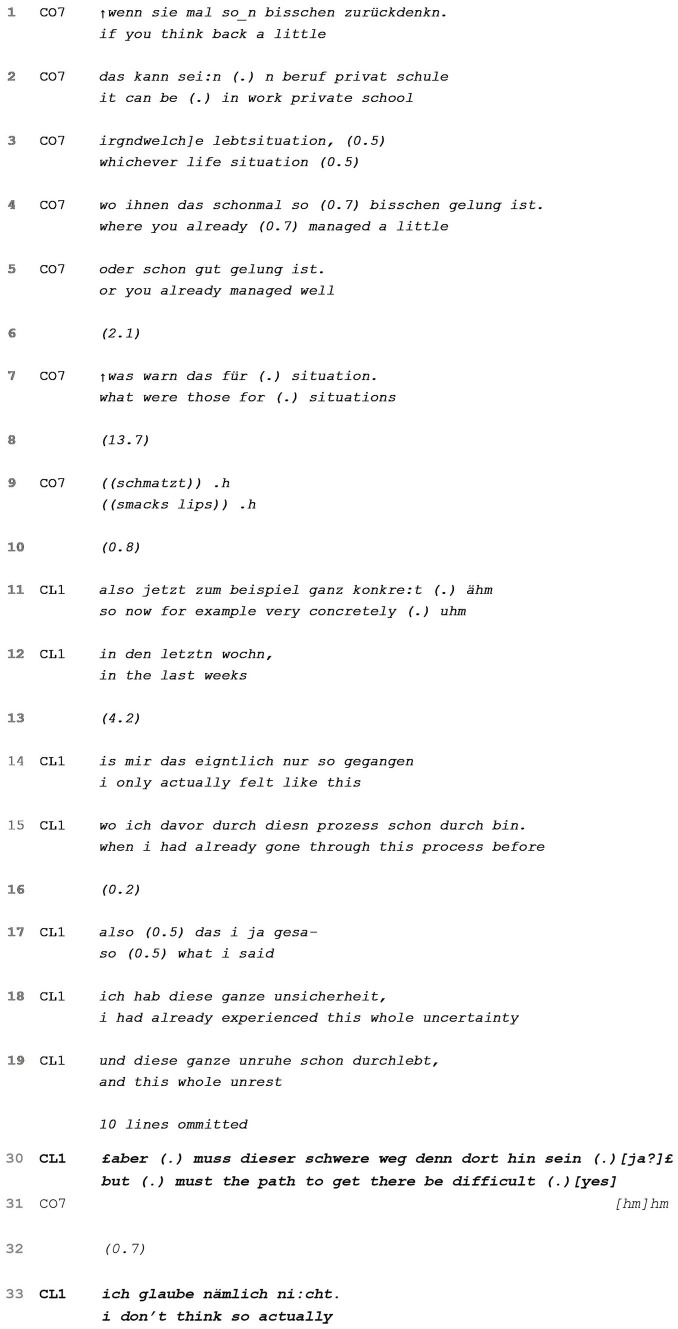

Although the formulation of the question in the past tense suggests that the client should look into past memories or situations going as far back as to “school” (lines 2–3), after a long silence (line 8), the client disregards this and chooses to focus on something recent and “very concrete” (line 11), namely thinking back on the “last weeks” (line 1434). The rising final contour as well as the ensuing gap in line 13 leaves space for the coach to correct this course of action, which he does not. The client then continues that such situations (i.e., in which she felt self-confident and serene) have occurred, but only *after* she had experienced the undesired pattern of second-guessing herself and feeling insecure (lines 14–19). With a smiling voice, she orients to the inadequacy of her response in line 30 “but (.) must the path to get there be difficult, yes?” She thereby reveals that she does not see these situations as ones where she “managed well” and invites the coach to agree with her using a question tag. In doing so, she does not provide the sought for example situation, but refocuses away from the positive course of action initiated by the coach and brings in an ambivalent stance. Though the idealized state is not completely new to her, it is closely linked with the problematic pattern she had previously described. The client thus returns to the underlying problem. Still, she re-orients to the solution talk in the end when stating with certainty that the difficult path is not necessarily a prerequisite, thus veering toward further solution exploration.

#### 5.2.2 (Partially) answering but refocusing

This category may be realized in a multitude of manners and forms (see Humă et al., 2023). Though clients first provide an answer here, it usually involves the client qualifying said answer, thereby limiting their agreement with the proposition, or answering the question in a ‘pro-forma’ manner, but then pursuing their own course of action (i.e., ‘refocusing’). This positions the coach’s question as (to some degree) inadequate or irrelevant for the client’s concern or current state of mind. Extract 8 exemplifies the latter form. The sequence takes place during the third session, in which the client informs the coach that she will soon be taking on a new position and thus needs to resign. Throughout the session, the client repeatedly topicalizes her guilty conscience. The excerpt starts just after a formulating (Heritage and Watson, 1979) passage by the coach in which she summarizes the client’s fear that her colleagues will accuse her of letting them down. This fear is what is anaphorically referred to in the coach’s use of “something like this” in the contextualizing preface (“if you hear something like this”) to her wh-question in the conditional mode (Extract 8, lines 1–3).

**EXTRACT 8:** Answering but refocusing.

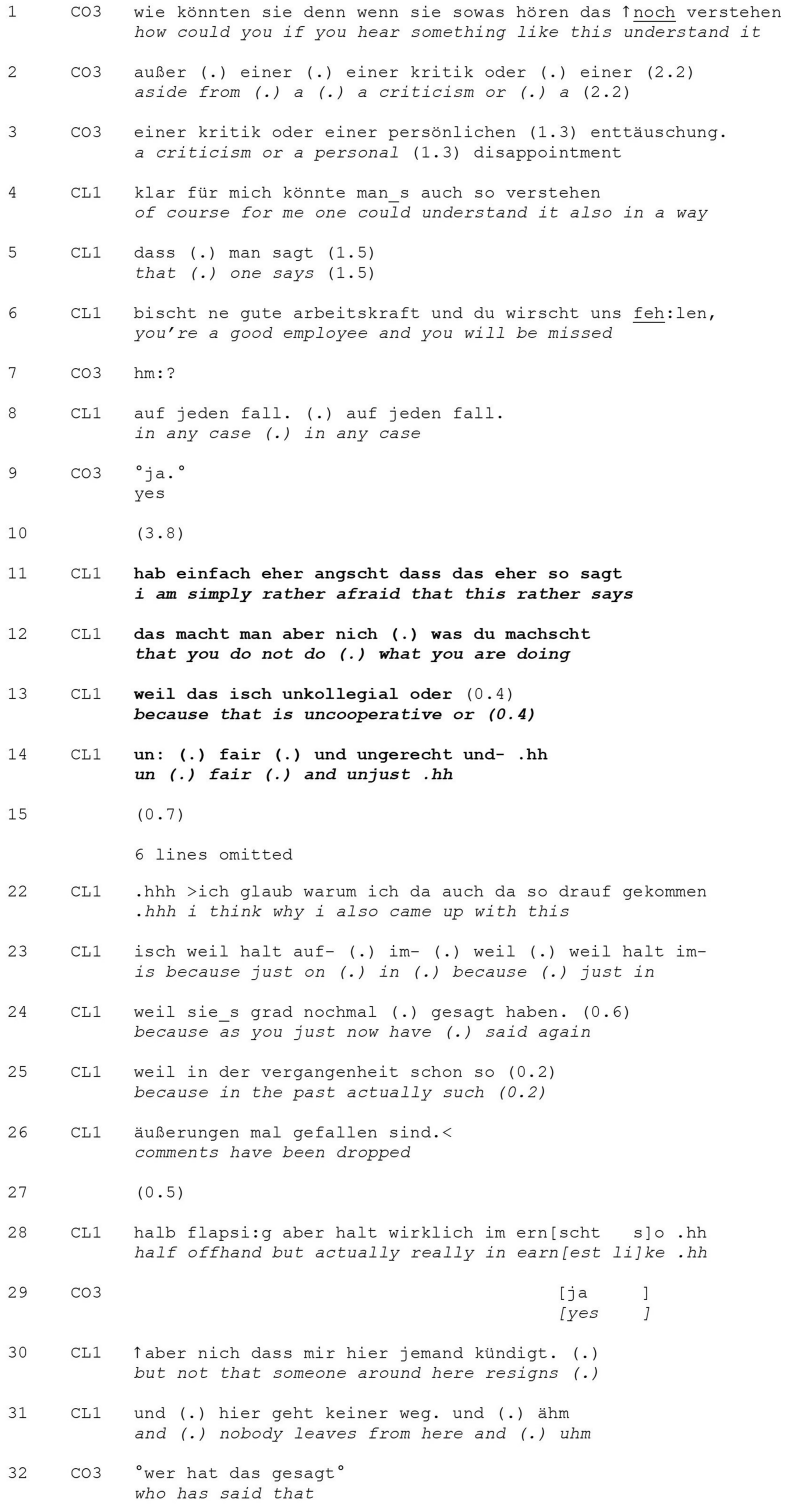

The question aims at transforming a negative perception– thus making a positive understanding of possible accusations from the side of the client’s colleagues relevant for the client’s answer. Explicitly relating her response to her situation (“for me”), the client does provide this in lines 4 to 6. She frames these possible understandings as obvious or self-evident with the use of evidential markers such as “of course” (line 4) and the double “in any case” (line 8), thus indexing the question as not directly relevant for the client’s situation (see, e.g., Stivers, 2018). Later on (starting in line 11), it becomes clear that the client only ostensibly (in a *pro-forma* manner) agreed with the suggested course of action, i.e., a change in perspective, while the rest of her reaction clearly disaffiliates with it.

The client’s answer is weakly ratified by the coach (line 9), who does not claim speaking rights. After a 3.8 s gap (line 10), the client continues with her turn, and signals that the course of action suggested in the coach’s wh-question, i.e., changing a negative understanding into a positive one, does not concur with her interpretation of the situation, which she then goes on explaining. In line 11, she refocuses on her fears, using “rather” (twice) to frame her own negative understanding and her colleagues’ positioning of her behavior as “uncooperative,” “unfair” and “unjust” (lines 12–14) as the more plausible interpretation of the situation. By doing so, she asserts primary rights to her feelings and preoccupations and again externalizes her concern, contrasting her position with that of the coach, who had implied that this was simply a matter of changing the client’s perspective. The client supports her own argument by launching an account of her own behavior (data not shown), and then adding a possible explanation for her fear, namely that such comments have already been made (“because actually in the past such comments have been dropped” in lines 25–36) and that these had been made “actually really in earnest” (line 28). The client thus resists a change in perspective at this point in the coaching process, which would allow for an alternative (affective) evaluation of having to leave her current job. The client rather initiates a loop, which suggests a necessity for further problem orientation rather than the solution-focus introduced by the coach. Nevertheless, the client is open to exploring her feelings and personal experiences.

Extract 9 shows another design of how clients answer but refocus. Prior to the extract, coach and client have been discussing ideal career paths. At some point, the client mentions in passing that self-employment could be an option for her, which prompts the coach to request stance-taking regarding this self-employment goal (lines 1–5).

In the initial formulation of her scaling question, the coach uses the adjective “strong” (line 3) as a basis for the client’s qualification of being self-employed. This presupposition reveals itself to be false and is later on explicitly refuted by the client (line 7). Following the client’s silence in line 4, which indicates upcoming misalignment and a dispreferred response, the coach formulates a new version of her question, this time presupposing that the wish might feel “good” (line 5). After another silence of 3.5 s in line 6 and a turn-initial acknowledgement token, the client refutes the idea of the “wish” to be “strong”. After a false start, the client then accounts for the rationale behind naming self-employment as a viable – indeed “attractive” (line 9) – option, namely flexibility, which she qualifies as “very important” to her (line 13). The client then returns to the coach’s request(s), and finally provides a dispreferred answer, a numerical value of “three or four” (line 15). The coach again prompts the client to elaborate with a continuer (Schegloff, 2007) in line 17. In her elaboration, the client completely refocuses away from the initial question, explicitly mentioning this in lines 23–24 (“it was not a self-employed position”). By recounting her impressions of a recent job interview, the client qualifies what she means by flexibility: on the one hand, flexibility is what she considered an attractive quality of self-employment; on the other hand, flexibility should not mean a complete absence of framework in an organization. The client’s refocusing is thus twofold: first, she refocuses from the self-employment status as something that she wishes for herself, accounting for her mentioning only because the flexibility it suggests is a positive characteristic for her. Secondly, the client refocuses from the hypothetical future addressed by the question toward her actual, present experiences, thus partly turning away from the solution-orientation yet still evincing aspects that should be integral characteristics of her future place of employment.

## 6 Discussion

Our study has focused on clients’ responsive actions which show resistance in answering within 82 wh-questioning sequences from business coaching overall and within 26 sequences corresponding to more active, agentive, and/or explicit resistive actions. We now discuss these findings by drawing on Muntigl’s (2023) concept of *moving against* (in contrast to *moving away* from) or ‘opposing’ the coach’s suggested course of action, and explain how a third form of resistance has emerged in the data, which we have termed *moving around* or ‘refocusing’. Moreover, we draw on Humă et al. (2023) concepts of the levels of resistance, the degree of explicitness in the realization of resistance (face threat) and the clients’ agency (passive/moving away vs. active/moving around and moving against; see also Koenig, 2011; Hollander, 2015). Finally, we explore how clients’ resistive practices may relate to the helping format business coaching.

We found that clients actively and explicitly *move against* the constraints and even the asking of questions (i.e., the prior action itself) in that they a) disagree with the question’s agendas or presuppositions, b) complain about having to answer questions, and c) refuse to answer altogether. Clients may misalign with, i.e., resist, the formal, topical, and agenda constraints as formulated in the coach’s wh-question. This involves topicalizing problems with answering the initial question, though clients often retroactively modify the question’s terms or agenda. Though explicit in its display of resistance, this may allow for the progressivity of the interaction (at least to some extent). In complaining, clients misalign by offering unfitted responses to the question. They substitute the fitted second pair part with their own new initiating action which requires attending to by the coaches and takes precedence over the initial question. In complaining, clients endanger their relationship with the coaches, as this represents an active face threat to the coach. In these cases in our data, the client thus softens this threat with prosocial elements in the aftermath, yet still declines to answer in the responsive turn. Moreover, clients’ active and explicit / plain refusal to answer the question constitutes a general rejection of the task (i.e., misalignment) and course of action (i.e., disaffiliation) set by the coach. In doing that, clients move against their coaches and the working alliance by openly claiming that the course of action is not worth consideration. This constrasts with ‘remaining silent’ – the lack of reaction remains open to interpretation and can thus be managed in a manner which allows for the safeguarding of face for both coaches and clients. *Moving against* thus constitutes the most explicit and challenging forms of resistance.

Additionally, we found that clients may effectively sidestep, bypass, or circle around courses of action, question constraints, or problematic elements thereof. This allows for the clients’ advancing of their own agenda and needs, suggesting an alternative (and competing) course of action to that of the coach and possibly a third category: *Moving around*. In our data, we found that refocusing responses represent more implicit forms of resistance to the question (as in ‘not answering and refocusing’). At the same time, they also display different degrees of cooperation (e.g., first providing an answer and then introducing an alternative course of action). To soften the impact of disaffiliation, clients generally design their turns using typical mitigating strategies. At the same time, while misaligning with the original question and its implications, adapting, i.e., ‘refocusing’, in itself may denote a willingness to respond in a manner that is productive, i.e., that cooperates with the overall aims of the coaching project if not the question in its particulars (Pomerantz, 2021). This, in contrast, indexes client affiliation.

The involvement and agency of coaching clients is further supported by the fact that a large part of the 26 instances of client resistance in our data functions as *moving around,* but still generally acts in a productive manner for the coaching project. While clients may indicate further need for problem orientation (see Extracts 6, 8) thereby opposing solution-oriented courses of action as introduced by the coach for the time being, in contrast to MacMartin’s (2008) findings, this does not represent a general refusal to optimistic content or solutions *per se*. Rather, clients agentively engage in further problem exploration or explication as the currently more relevant course of action, thus claiming responsibility for their own change process. Additionally, clients may also work to introduce an alternative solution or topic thereby orienting to the overall solution- and goal-orientation of the coaching interaction (see Extracts 7, 9). Stivers’ work on transformative answers qualifies this response type by clients as enacting “significant autonomy” (Stivers, 2022, p. 151, see also Stivers and Hayashi, 2010). We found this to be true for our practices doing *moving around*, too. Indeed, clients have the possibility to highlight their epistemic and deontic authority over what constitutes a good path and/or a good outcome in their own situation (see also Muntigl et al., 2020a and Smoliak et al., 2022 on negotiation of authority in psychotherapeutic interactions).

**EXTRACT 9:** Answering but refocusing.

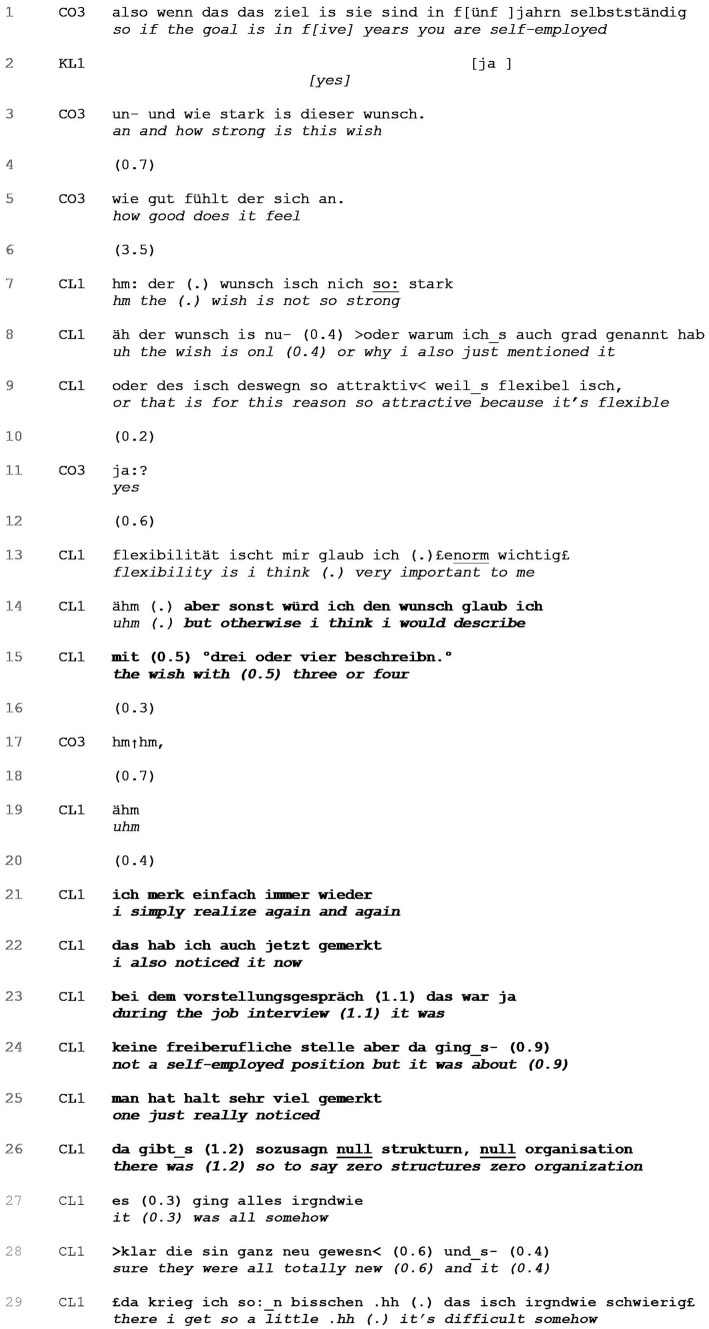

## 7 Limitations of the study and outlook

The present work has focused on only one type of questioning sequence in business coaching, meaning that further research will be needed to explore resisting practices to polar (both interrogative and declarative forms) and alternative questions. The focus has not been on resistance management by coaches. Moreover, we have not explored non-vocal resisting practices, in which clients provide an answer, for instance, but indicate via gaze, body movements, gestures, etc. that the question may be problematic. Additionally, in light of the apparent readiness of clients to further the coaching project, research into the closely-linked phenomenon of same-turn delaying but answering (or responding productively) to questions in coaching should be considered. By this we mean that, via various interactional resources such as humor, long gaps, turn-initial accounts, no-access responses or evaluations of the question as difficult, etc., clients may initially withhold an answer but follow up on this delay by (tentatively) formulating an answer within the same turn (and thus not blocking the progressivity of the sequence). This could lead to valuable insights into the concept of ‘reflection’, where the delay can be interpreted as an indication that clients need more time to think (indeed, reflect) to respond to the question in a productive manner.

## Data availability statement

The original contributions presented in the study are included in the article/supplementary material, further inquiries can be directed to the corresponding author.

## Ethics statement

Written informed consent was obtained from the individual(s) for the publication of any potentially identifiable images or data included in this article.

## Author contributions

FD and MF examined the corpus for the occurrence of wh-sequences displaying resistive actions and were responsible for the analysis, supported by PM. FD, MF, PM, and E-MG discussed the categories. All authors contributed to the article, writing and revising of the final manuscript, designed the study together, and approved the submitted version.

## Footnotes

^1^In the entire QueSCo project data (see below), we identified 3,023 question and questioning sequences across 14 coaching processes and 50 sessions with questions amounting to 16% and questioning sequences amounting to 83% of the entire transcribed data.

^2^In a sample of 9 different processes (27 sessions) from the QueSCo project data (see below), and from a total number of 1.914 questions asked by coaches, 1.018 are wh-questions. This amounts to 53,2%, while the remaining 46,8% are distributed among polar interrogative questions, alternative questions, and declarative questions.

^3^Questioning Sequences in Coaching (I 4990-G) is funded by the Austrian Science Fund (FWF), the German Research Foundation (DFG) and the Swiss National Research Foundation (SNF) and runs from 2021 to 2024. The project aims to shed light on the nature of questioning sequences in business coaching as well as their change-inducing potential, combining linguistic and psychological perspectives and using mixed-methods to do so. More information regarding the corpus and project can be found on the official project website: https://questions-in-coaching.aau.at/en/.

^4^Such strong personal involvement on the part of clients was also found in Spranz-Fogasy and colleagues’s comparative study on example requests in psychotherapeutic and coaching interactions (2019). Indeed, they evinced that clients often did not need to be prompted at all by the coaches and tended to provide examples of their own volition.

^5^Such an explicit resistive move by the client seems to derive and culminate from prior occurrences of more implicit client resistance. Prior to the sequence of Extract 1, the client had shown tendencies of refocusing to avoid talking about himself (see below for refocusing; see also Fenner et al., 2022 on verbosity as a form of resistance). It is by refusing to answer a question positing a direct connection to his concern that the client’s resistance becomes explicit (see also Clark et al., 1994 on the trajectory from implicit to explicit resisting). This tendency marking a trajectory from implicit to explicit resistance was not generally observed in our corpus.

^6^A look at the video recording reveals that they are smiling at each other in that moment.
